# Hsa_circ_0008870 suppresses bone formation of growth plate through inhibition of miR-185-3p/ MAPK1 axis in idiopathic short stature

**DOI:** 10.3389/fbioe.2022.1022830

**Published:** 2022-10-11

**Authors:** Zhiwen Wu, Jinghong Yuan, Jiantian Li, Zhi Du, Ming Yin, Xigao Cheng, Xijuan Liu, Jingyu Jia

**Affiliations:** ^1^ Department of Orthopaedics, The Second Affiliated Hospital of Nanchang University, Nanchang, Jiangxi, China; ^2^ Institute of Orthopedics of Jiangxi Province, Nanchang University, Nanchang, Jiangxi, China; ^3^ Institute of Minimally Invasive Orthopedics, Nanchang University, Nanchang, Jiangxi, China; ^4^ Jiangxi Key Laboratory of Intervertebral Disc Disease, Nanchang University, Nanchang, Jiangxi, China; ^5^ Department of Paediatrics, The Second Affiliated Hospital of Nanchang University, Nanchang, Jiangxi, China

**Keywords:** circular RNA, growth plate, idiopathic short stature, miR-185-3p, MAPK1

## Abstract

Idiopathic short stature (ISS) is the most common clinical cause of the short stature with an unclear aetiology and a lack of effective treatment. Circular RNAs have been shown to play a significant regulatory role through various signal transduction pathways in a variety of diseases in recent years. However, the role of circular RNAs on ISS is not yet well-understood and requires a special attention. The differentially expressed circular RNAs were screened by microarray chip analysis, and RT-qPCR was used to verify the expression of hsa_circ_0008870 in ISS patients. Subsequently, *in vitro* and *in vivo* experiments were conducted to determine the biological functions of hsa_circ_0008870 in ISS. The authors first confirmed that hsa_ circ_0008870 was downregulated in ISS children. Meanwhile, we also observed that the downregulated hsa_circ _0008870 significantly inhibited chondrocyte proliferation and endochondral ossification *in vivo* and *in vitro*. Mechanistically, hsa_circ_0008870 regulates MAPK1 expression by sponge miR-185-3p. This mechanism of action was further verified through rescue experiments. Finally, the authors revealed that the silencing of hsa_circ_0008870 induces low expression of MAPK1 by impairing the sponge action of miR-185-3p, thereby inhibiting chondrocyte proliferation, hypertrophy, and endochondral ossification, which results in a short stature phenotype. In addition to these, we also observed an interesting phenomenon that upregulated of miR-185-3p can in turn inhibit the expression of hsa_circ_0008870 in chondrocytes. This suggests that hsa_circ_0008870 could potentially serve as a therapeutic target for the treatment of ISS.

## 1 Introduction

Short stature consistently remains the most common reason for referral to a pediatric endocrinologist (Collett-Solberg et al., 2019). Idiopathic short stature (ISS) accounts for 60%–80% of cases with short stature in children (Cohen et al., 2008). ISS is characterized with a height below the two standard deviations or the third percentile of the normal reference levels for the same ethnic group, gender and age, without chronic systemic, endocrine, nutritional, skeletal diseases or chromosomal abnormalities (Lee et al., 2020). To date, no breakthrough has been made in the field of treatment to improve height in ISS children due to the uncertain etiology.

Recombinant human growth hormone (rhGH) was first introduced in 1985 to increase height in short children (Bryant et al., 2007; Ross et al., 2015). Since 2003, rhGH has been used for the treatment of ISS (Grimberg et al., 2016; Wu et al., 2020). However, as all known, the children with ISS are not deficient in growth hormone (Lee, 2006)**.** Therefore, the efficacy of the rhGH to treat ISS is controversial so far (Pedicelli et al., 2009). Although Paltoglou et al. reported that ISS children who received rhGH treatment increased significantly in height after 1 year, and Barton et al. reported that increasing the dose of rhGH could further improve the height growth of patients (Barton et al., 1995; Paltoglou et al., 2020), Kamp et al. study found that epiphyseal closure was advanced in ISS children, the growing period was greatly shortened and no improvement in height at the termination of growth after the treatment of rhGH (Kamp et al., 2002). In addition, several studies have reported many potential risks to children and complications during rhGH therapy, including cancer, scoliosis, slipped capital femoral epiphysis, idiopathic intracranial hypertension, carbohydrate metabolism disorders, and hypertension, suggesting that rhGH therapy may require stricter consideration and regulation (Vyas and Menon, 2021). Worse yet, rhGH treatment for patients with ISS proved extremely expensive, expected to spend $52,000 per inch of height gain (Lee et al., 2006). The main reason for the difficulty of diagnosis and poor treatment of ISS disease is that the pathogenesis of ISS is still unclear. Therefore, elucidating the molecular mechanisms of ISS initiation and progression is of great significance for improving the diagnostic and therapeutic efficiency of ISS.

As a special type of endogenous non-coding RNAs, circular RNAs (circRNAs) are a research hotspot in RNA biology (Zhao et al., 2019). Unlike linear RNA, circRNAs have no 5′caps and 3′polyadenylic acid tails (Chen and Yang, 2015). With their closed-loop structure, circRNAs can avoid degradation by RNase enzymes. Hence, its expression is more stable (Greene et al., 2017; Zhao et al., 2019). Circular RNAs have been shown to play a significant regulatory role through various signal transduction pathways in a variety of diseases in recent years (Qu et al., 2015). For instance, CircACTN4 was demonstrated to be upregulated in ICC (intrahepatic cholangiocarcinoma) patients, acting as a molecular sponge of miR-424-5p and interacting with YBX1, to transcriptionally activate FZD7, thereby promoting proliferation and metastasis of ICC (Chen et al., 2022). CircFndc3b enhanced the expression and signal of vascular endothelial growth factor-A by interacting with the RNA binding protein fused in sarcoma to promote cardiac function and remodeling after myocardial infarction (Garikipati et al., 2019). Additionally, circSERPINE2, acting as the sponge for miR-1271, suppressed human chondrocytes apoptosis and promoted the synthesis of extracellular matrix, thus inhibiting its apoptosis and delaying the progress of osteoarthritis (Shen et al., 2019). Related studies showed that circRNAs have been tightly associated with the occurrence and development of heart vascular, digestive system diseases, malignant tumours, osteoarthritis and neurological diseases (Qu et al., 2017). To determine the role of circRNAs in the molecular mechanisms of ISS, researchers identified differential expression of circRNAs in the peripheral blood of patients with ISS *versus* healthy controls. According to the outcomes of the circRNAs microarray, 145 differentially expressed circRNAs were screened (83 upregulated and 62 downregulated) (Liu et al., 2020; Liu et al., 2021). In subsequent studies, it was verified that circANAPC2 and hsa_circularRNA_0079201 can serve as potential targets for treatment of ISS (Liu et al., 2020; Liu et al., 2021).

To further evaluated the role of other circRNAs in pathogenesis of ISS, hsa_circ_0008870 was detected to be downregulated in ISS, and its downregulation was subsequently confirmed using RT-qPCR. Then, it was observed that the hsa_circ_0008870 downregulation suppressed the expression of MAPK1 *via* upregulating miR-185-3p, and finally inhibited chondrocyte proliferation, chondrocyte hypertrophic differentiation and endochondral ossification *in vitro* and *in vivo*. Interestingly, the authors observed that the upregulation of miR-185-3p can further repress the hsa_circ_0008870 expression *via* a positive feedback loop.

## 2 Materials and methods

### 2.1 Patient information and specimen acquisition

64 idiopathic short stature (ISS) patients comprised the experimental group. These patients received treatment in the Second Affiliated Hospital of Nanchang University (Nanchang, China) from August 2018 to August 2020.64 healthy children comprising the normal group, received physical examination during the same period. The conditions for patient enrollment in this study were consistent with our previous studies (Liu et al., 2021).

The ISS patients group comprised of 29 males and 35 females at the estimated average age of 9.22 ± 1.87 years with the average height of 121.38 ± 11.40 cm. The normal group comprised of 38 males and 26 females at the average age of 9.16 ± 1.91 years. The estimated average height for the control group was 130.24 ± 10.87 cm (Table 1). All blood samples were frozen immediately in liquid nitrogen after collection to prevent the degradation of biomolecules and contamination in samples. All samples were stored in a refrigerator at -80 °C until use. Blood samples from four ISS patients and four normal children were used for circRNAs microarray chip analysis, while all 64 pairs of blood samples were used for quantitative Real-Time PCR (RT-qPCR) to quantify the circRNAs expression.

**TABLE 1:** The information of ISS patients and normal control individuals (NC).

Group	ISS	Normal controls
Sex		
Male	29	38
Female	35	26
Mean age ±SD, years	9.22 ± 1.87	9.16 ± 1.91
Age range, years	4–12	4–12
Mean height ±SD, cm	121.38 ± 11.40	130.24 ± 10.87
Height range, cm	84–139.6	95.7–146.5

ISS, idiopathic short stature.

### 2.2 Cell culture

Human chondrocyte cell line C28/I2 was sourced from Procell Life Science & Technology Co., Ltd. In Wuhan, China. The complete medium consisted of 89% Dulbecco’s modified Eagle medium (DMEM/F12; Gibco, United States), supplemented with 10% fetal bovine serum (FBS; Gibco, United States) and 1% penicillin-streptomycin (Invitrogen). Cells were incubated in a sterile incubator at 5% CO_2_ saturation and 37°C constant temperature.

### 2.3 Cell transfection

Small interfering RNAs (siRNAs) for circ-0008870 (designated as si-circ-0008870) and miR-185-3p mimics, miR-185-3p inhibitor and their negative control (NC) were obtained from RiboBio (Guangzhou, China). According to the instructions of the manufacturer, transient transfection was conducted employing Lipofectamine 3,000 (Invitrogen).

The lentiviral of sh-circ-0008870, circ-0008870-overexpressing and corresponding NC were constructed by the GeneChem (Shanghai, China). When chondrocytes confluence reaches about 30%–40%, then transfection was performed. Stable transfected cells were sifted with puromycin.

### 2.4 RT-qPCR

The chondrocytes were lysed following the instructions of the Trizol reagent RNA extraction kit (Invitrogen, United States) and total RNA was extracted. The RNA was reverse transcribed using the PrimeScript RT Master Mix Kit (Takara). Quantitative PCR was executed utilizing a real-time PCR kit (Takara) and the specific primers. For primers, see Supplementary Table S1. Each experiment was conducted in triplicates. The endogenous controls were GAPDH and U6 small nuclear RNAs. The relative expression level of RNA was analyzed by the 2^−ΔΔCt^ method.

### 2.5 Western blotting

Cells were collected by cell scraping. RIPA lysis buffer rich in protease inhibitors (Beyotime, China) was applied to extracted the proteins. BCA protein quantification kit (Beyotime, China) was applied to detect the proteins concentrations following the instructions of the manufacturer. Proteins were separated by electrophoresis using SDS-PAGE (Beijing Biosynthetic Biotechnology, China) and transferred to a polyvinylidene fluoride (PVDF) membrane (Bio-Rad, United States). The PVDF membrane was put into 5% skimmed milk powder blocking solution (Solarbio, China), and sealed for 2 h at room temperature. The sealed PVDF membrane was incubated with the anti-OCN (1:1000; Abcam, ab133612), OPN (1:1000; Abcam, ab214050), RUNX2 (1:2000; Abcam, ab76956), Collagen X (1:1000; Abcam, ab182563), MAPK1 (1:5000; Abcam, ab265600), GAPDH (1:2500; Abcam, ab9485), MEF2A (1:1000; Abcam, ab109420) overnight at 4°C.According to the source of the primary antibody, the secondary antibody was diluted with TBST solution and the PVDF membrane was put into the corresponding secondary antibody. An ultra-sensitive ECL chemiluminescence kit was applied to expose the membrane. The chemiluminescence of the protein bands was quantified by ImageJ software.

### 2.6 Flow cytometry

The cell cycle detection was performed using the KeyGEN Cell Cycle Kit (China). Briefly, samples were centrifugated to collect the cells, which were rinsed with PBS. The collected cells were fixed with ice-cold 70% ethanol for 4 h at 4°C. RNase A and propidium iodide (PI) were mixed and kept at RT without exposure to sunlight for 30 min. Flow cytometry was conducted to detect DNA, the red fluorescence was recorded at the excitation wavelength of 488 nm. Flow cytometry (Becton Dickinson, United States) was used to analyze the cell distribution ratios in each cell cycle stage.

### 2.7 Cell proliferation

Cell proliferation was detected using the Edu Cell Proliferation Assay Kit (RiboBio, China) with the protocol of manufacturer. In 12-well plates, chondrocytes were seeded and incubated for 24 h at 37°C. The cells transfected with siRNA or lentivirus were cultured in the medium containing 50 µM Edu for 2 h. After fixing the cells, they were stained with Apollo and were observed under laser scanning confocal microscope (Nikon Instrument, Japan). Data quantitation was done by counting the Edu positive (red) and Hoechst 33342 positive cells (blue). The proliferation rate was calculated by the ratio between the Edu positive *vs* Hoechst 33342 positive cells.

### 2.8 Luciferase reporter assay

Luciferase reporter gene vectors (circ-0008870-wt and MAPK1-wt; circ-0008870-Mut and MAPK1-Mut) (GenePharma) were constructed. Lipofectamine 3,000 (Invitrogen, United States) was used for co-transfection of the cells with circ-0008870-wt, circ-0008870-mut, MAPK1-wt, MAPK1-mut, miR-185-3p mimics or negative control (NC). After culturing for 48 h and the luciferase activity was detected using the Dual-Luciferase Assay System (Promega, United States).

### 2.9 *In situ* hybridization

The has-cir-008870 was labelled with Cy3 whereas miR-185-3p was with FAM. DAPI (4′,6-diamino-2-phenylindole) was also used for staining. All staining procedures were done following the instructions in the fluorescence *in situ* hybridization kit (FISH) kit (GenePharma, China). Imaging was conducted by laser scanning confocal microscope (Nikon Instrument, Japan).

### 2.10 RNase R treatment assay

Chondrocytes were collected and Trizol reagent was added to lyse and extract RNA. RNase R was added to the mixture, which was incubated for 30 min at 37°C. Additionally, the expression of hsa_circ_0008870 and the corresponding linear RNAs were detected by RT-qPCR.

### 2.11 Alkaline phosphatase (ALP) and calcium staining

ALP staining solution (azo-coupling method) kit (Solarbio, China) was used for ALP staining. Chondrocytes were seeded in 12-well plates and mixed with ALP fixative solution for 3 min at RT. Added the ALP incubation solution (AS-BI: FBB = 1:1) dropwise and incubated the mixture in the dark for 20 min. Chondrocytes were counterstained with nuclear fast red staining solution for 5 min. Finally, viewing cells with a microscope (Nikon, Japan).

Chondrocytes were fixed in 4% paraformaldehyde and exposed to ultraviolet light for 30 min after Von Kossa silver solution staining. Then cells were mixed with Hypo solution for 2 min and hematoxylin staining solution for 25 min, Finally, eosin staining solution was added for 5 min. All staining procedures followed the instructions of the manufacturer (Solarbio, China).

### 2.12 Animal experiments *in vivo*

15 Sprague Dawley (SD) rats (3 weeks old) were divided into two groups and were injected with LV-sh-circ-0008870 and LV-NC lentiviral expression vectors, which were diluted with saline from the tail vein. All rats were fed for 6 weeks. During this period, the body length and weight were measured every 2 weeks. The Animal Ethics Committee of Nanchang University (Nanchang, China) gave the formal ethical approval for animal experiments.

Bone and cartilage specimens from the knee joints were collected and stored in 4% formalin. The samples were decalcified for 2months using 10% EDTA and then embedded in paraffin. The thickness of the sagittal section was 5 μm. Subsequently, slices are used for safranin O, immunohistochemistry (IHC) analysis and calcein staining. The specific methods refer to previous studies (Liu et al., 2022). RT-PCR was used to detect the expression levels of hsa_circ_0008870 and miR-185-3p. The expression levels of OCN, OPN, RUNX2, and collagen type X were detected by RT-qPCR and Western blot.

### 2.13 Statistical analysis

Experiments were conducted in triplicates and data are analyzed by GraphPad Prism 9.3 software. The experimental data are expressed as mean ± standard deviation (SD). Differences between groups were analyzed using student’s t test or one-way ANOVA. *p* values <0.05 indicated the statistically significant differences.

## 3 Results

### 3.1 Hsa_circ_0008870 is downregulated in ISS patients

**FIGURE 1:**
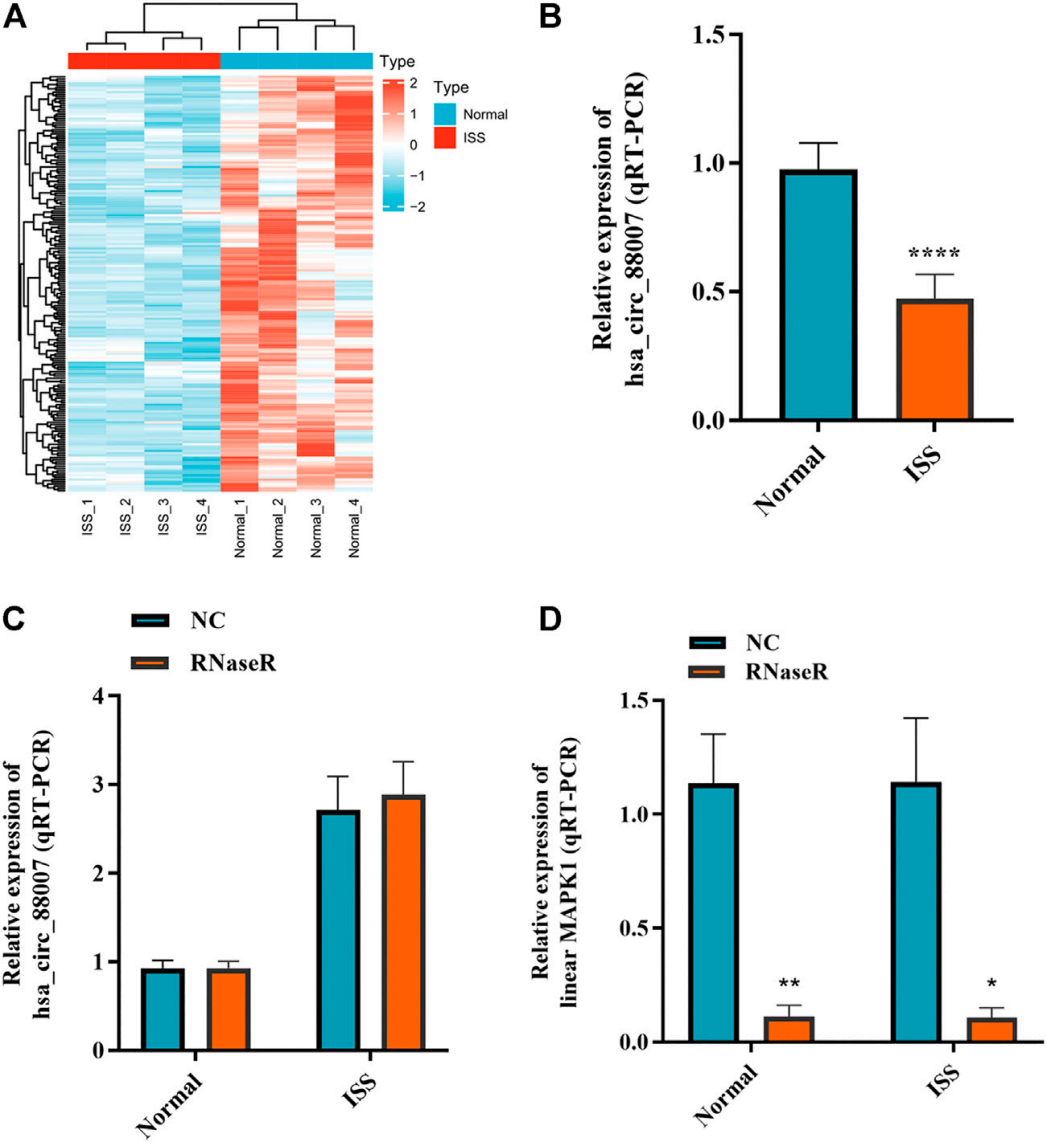
The expression of hsa_circ_0008870 in peripheral blood of ISS is downregulated. **(A)** Heatmap changes of downregulated circRNAs between the two groups. **(B)** Downregulated hsa_circ_0008870 in ISS patients were validated by RT-qPCR. **(C)** The expression of hsa_circ_0008870 was detected between the RNase R group and the normal control group. **(D)** The expression of liner MAPK1 was detected between the RNase R group and the normal control group. *p* > 0.05; *, *p* < 0.05; **, *p* < 0.01; ***, *p* < 0.001; ****, *p* < 0.0001.3.1.22.

The blood samples of four ISS and four normal children were used for circRNA microarray analysis. Downregulated circRNA expression profile was mapped by hierarchical clustering. The microarray result showed that 62 circRNAs were downregulated in ISS patients (Figure 1A). Because of the hsa_circ_0008870 expression downregulated significantly (*p* < 0.05, |logFC|-value > 2.0), hsa_circ_0008870 was selected as the candidate for further analyses in 42 ISS children and 42 normal children (Figure 1B). The expression levels of hsa_circ_0008870 were verified by RT-qPCR after RNase R was used to treat total RNA from ISS and normal groups (Figures 1C,D). These results revealed that hsa_circ_0008870 in ISS presented up-regulated expression compared with normal children instead of linear RNAs.

### 3.2 Knockdown of hsa_circ_0008870 suppresses chondrocyte proliferation and endochondral ossification

**FIGURE 2:**
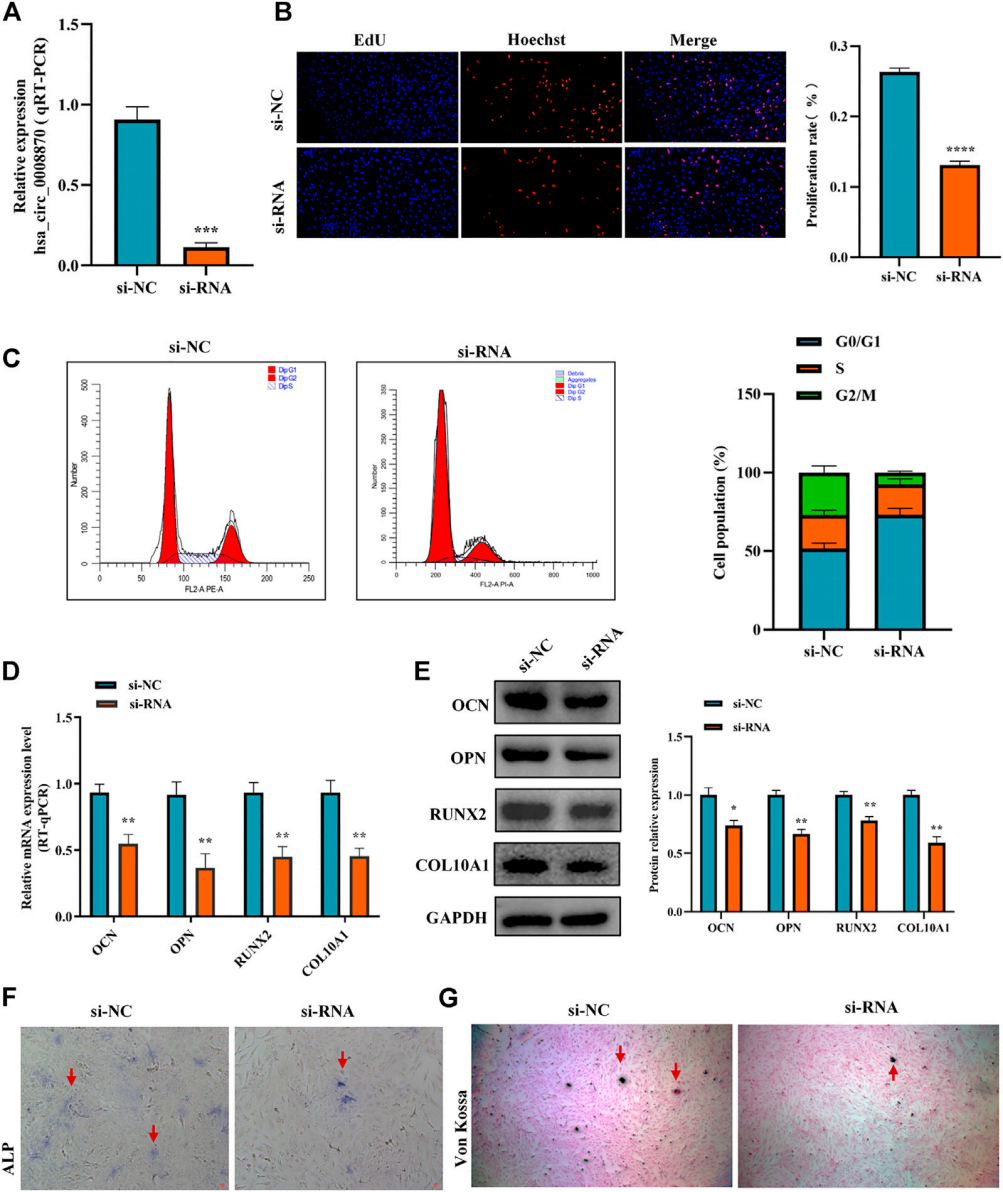
Silencing hsa_circ_0008870 inhibits chondrocyte proliferation, hypertrophy and endochondral ossification. **(A)** The expression of hsa_circ_0008870 in chondrocytes transfected with siRNA was detected by RT-qPCR. **(B)** Edu assays was used to detect changes in cell proliferation after knockdown of hsa_circ_0008870. **(C)** Flow cytometry assays indicate changes of cell cycle by knockdown of hsa_circ_0008870. **(D)** Following knockdown of hsa_circ_0008870, the mRNA expressions of OCN, OPN, RUNX2 and collagen type X were detected by RT-qPCR. **(E)** Following knockdown of hsa_circ_0008870, the protein expression levels of OCN, OPN, RUNX2 and collagen type X were detected by Western blot. **(F)** The activity changes of ALP were observed following knockdown of hsa_circ_0008870. **(G)** Von Kossa staining demonstrated changes in mineralization after knockdown of hsa_circ_0008870. P > 0.05; *, *p* < 0.05; **, *p* < 0.01; ***, *p* < 0.001; ****, *p* < 0.0001.3.1.23.

To figure out the function of hsa_circ_0008870 downregulation in ISS, the expression of hsa_circ_0008870 was silenced in human chondrocytes (Figure 2A). The Edu assays showed that the chondrocyte proliferation was markedly inhibited in the group of hsa_circ_0008870 downregulation compared with control group (Figure 2B). Meanwhile, flow cytometry analysis demonstrated that compared with the control group, a larger proportion of chondrocytes in the hsa_circ_0008870 down-regulated group were arrested in the G0/G1 phase (Figure 2C).

To clarify the effect of hsa_circ_0008870 downregulation on the hypertrophy, mineralization, and endochondral osteogenesis in chondrocytes, the expression of OCN, OPN, RUNX2 and collagen type X genes were determined using RT-qPCR and Western blot (Figures 2D,E). These results displayed that the expression of OCN, OPN, RUNX2, and collagen type X genes were significantly suppressed in the group of hsa_circ_0008870 downregulation. Furthermore, the ALP active nodules and mineralized nodules were decreased in the group of hsa_circ_0008870 downregulation using ALP activity analysis and Von Kossa staining (Figures 2F,G). These experimental outcomes indicated that silencing of hsa_circ_0008870 suppressed chondrocyte proliferation and endochondral ossification.

### 3.3 Hsa_circ_0008870 suppresses chondrocytes proliferation and endochondral ossification by modulating miR-185-3p

**FIGURE 3:**
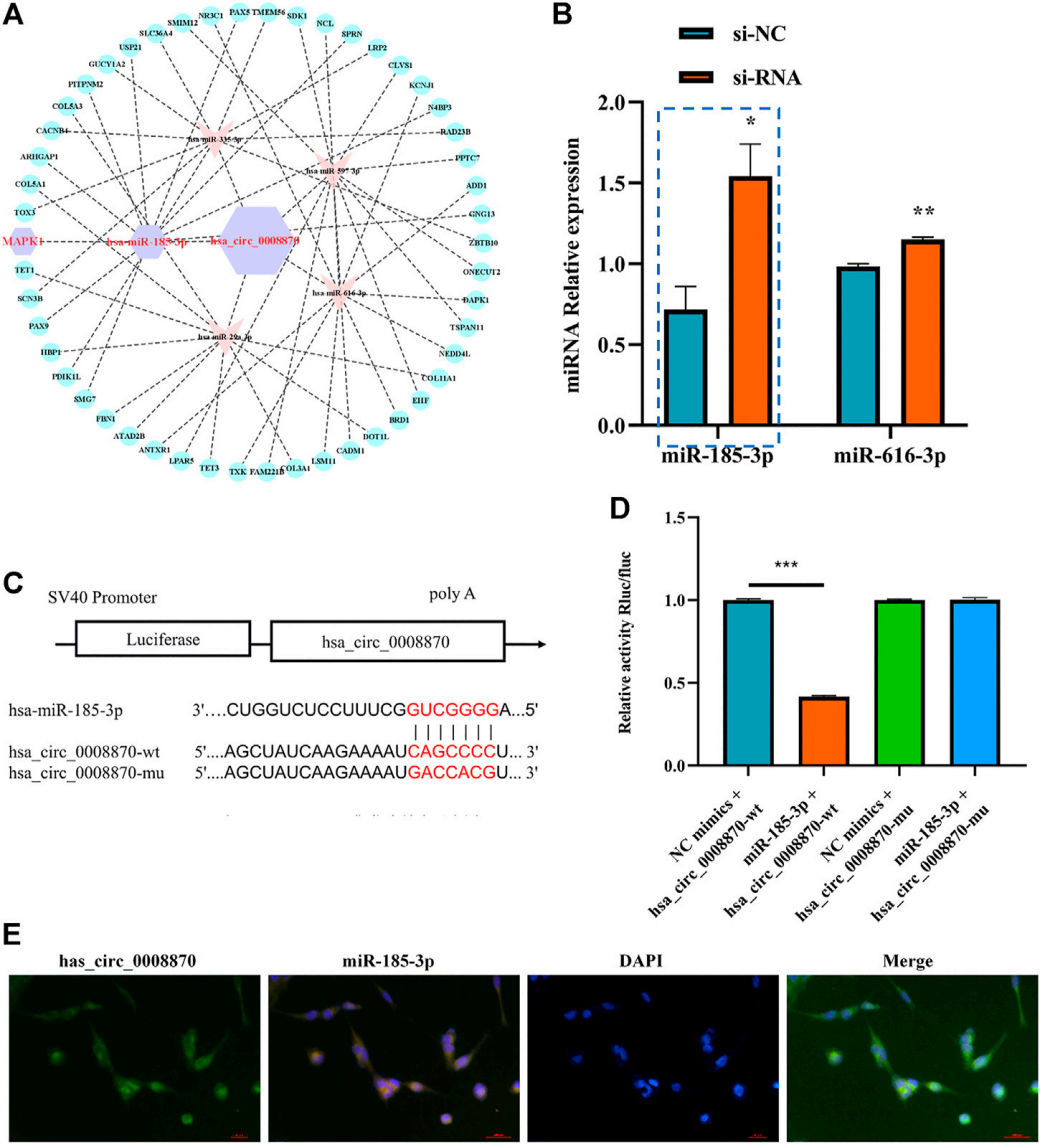
Hsa_circ_0008870 acted as an miR-185-3p sponge. **(A)** hsa_circ_0008870 targeted miRNAs-mRNAs network prediction. **(B)** The expression of predicted miRNAs in chondrocytes following downregulation of hsa_circ_0008870 expression. **(C)** The potential target binding sites between hsa_circ_0008870 and miR-185-3p. **(D)** Luciferase activity was repressed in human chondrocyte cotransfected with WT hsa_circ_0008870 and miR-185-3p mimics, while it was restored in cells cotransfected with Mut hsa_circ_0008870 and miR-185-3p mimics. **(E)** Fluorescence *in situ* hybridization proved the co-localization of hsa_circ_0008870 and miR-185-3p in human chondrocytes and mouse femoral growth plates. P > 0.05; *, *p* < 0.05; **, *p* < 0.01; ***, *p* < 0.001; ****, *p* < 0.0001.3.1.24.

To further elucidate the molecular mechanism of hsa_circ_0008870 in ISS, possible interactions between hsa_circ_0008870 and downstream miRNAs were predicted using Circinteractome (http://circinteractome.irp.nia.nih.gov) and manually, and to build the hsa_circ_0008870 targeted miRNAs-mRNA interaction network (Figure 3A). Subsequently, knockdown of hsa_circ_0008870 and RT-qPCR were used to validate the expression of all predicted miRNAs. As shown in Figure 3B, miR-185-3p and miR-616-3p were upregulated after hsa_circ_0008870 downregulation. The miR-185-3p was chosen as the candidate miRNA of hsa_circ_0008870 for further research due to the differential expression was the highest with a 2.41-fold (Figure 3B). The potential binding sites between this miR-185-3p and hsa_circ_0008870 are illustrated in Figure 3C. Afterwards, the luciferase results displayed that, compared with the control group, the fluorescence intensity of the hsa_circ_0008870-wt group decreased significantly, while the fluorescence intensity of the hsa_circ_0008870-mut group recovered (Figures 3C,D). This indicated that hsa_circ_0008870 could target and bind to miR-185-3p. Moreover, *in situ* hybridization proved the co-localization of hsa_circ_0008870 and miR-185-3p in human chondrocytes and mouse femoral growth plates (Figure 3E).

**FIGURE 4:**
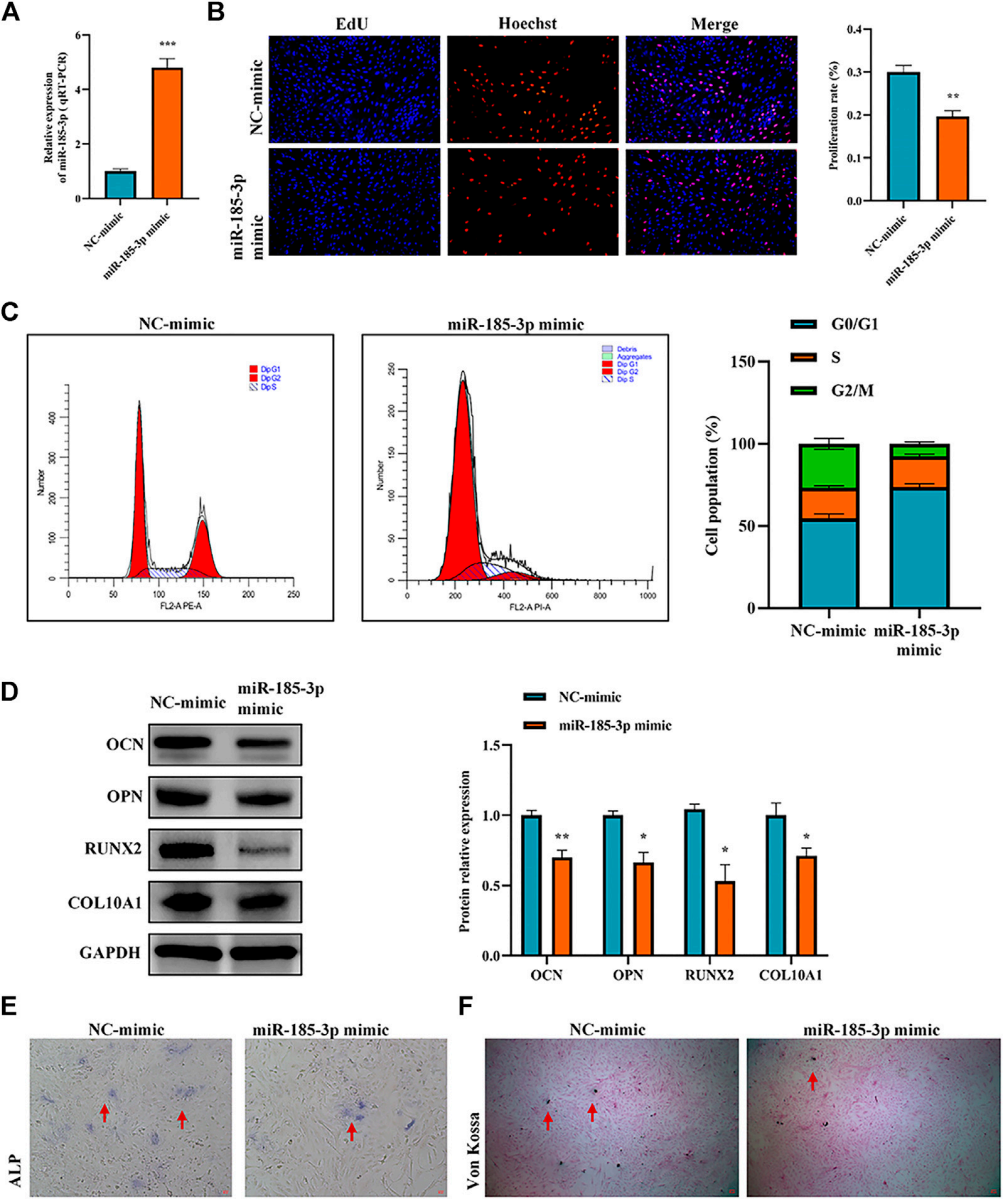
Upregulation of miR-185-3p suppresses chondrocytes growth and proliferation and cartilage osteogenesis. **(A)** The RNA levels of miR-185-3p in human chondrocytes transfected with miR-185-3p mimics were measured by RT-qPCR. **(B)** The effect of overexpression of miR-185-3p on chondrocyte proliferation was detected by Edu assays. **(C)** The effect of overexpression of miR-185-3p on chondrocyte cell cycle was detected by flow cytometry. **(D)** The protein expressions of OCN, OPN, RUNX2 and collagen type X were measured in miR-185-3p overexpressed chondrocytes. **(E)** ALP activity was measured in miR-185-3p overexpressed chondrocytes. **(F)** Von Kossa staining demonstrated changes in mineralization in miR-185-3p overexpressed chondrocytes. P > 0.05; *, *p* < 0.05; **, *p* < 0.01; ***, *p* < 0.001; ****, *p* < 0.0001.3.1.25.

The authors confirmed the function of miR-185-3p in chondrocytes. Firstly, we overexpressed miR-185-3p in chondrocytes (Figure 4A). Subsequent experiments revealed that overexpression of miR-185-3p obviously inhibited the proliferation level of chondrocytes (Figures 4B,C), decreased the expression levels of OCN, OPN, RUNX2 and collagen type X genes (Figure 4D), and reduced ALP activity and mineralized nodules (Figures 4E,F).

**FIGURE 5:**
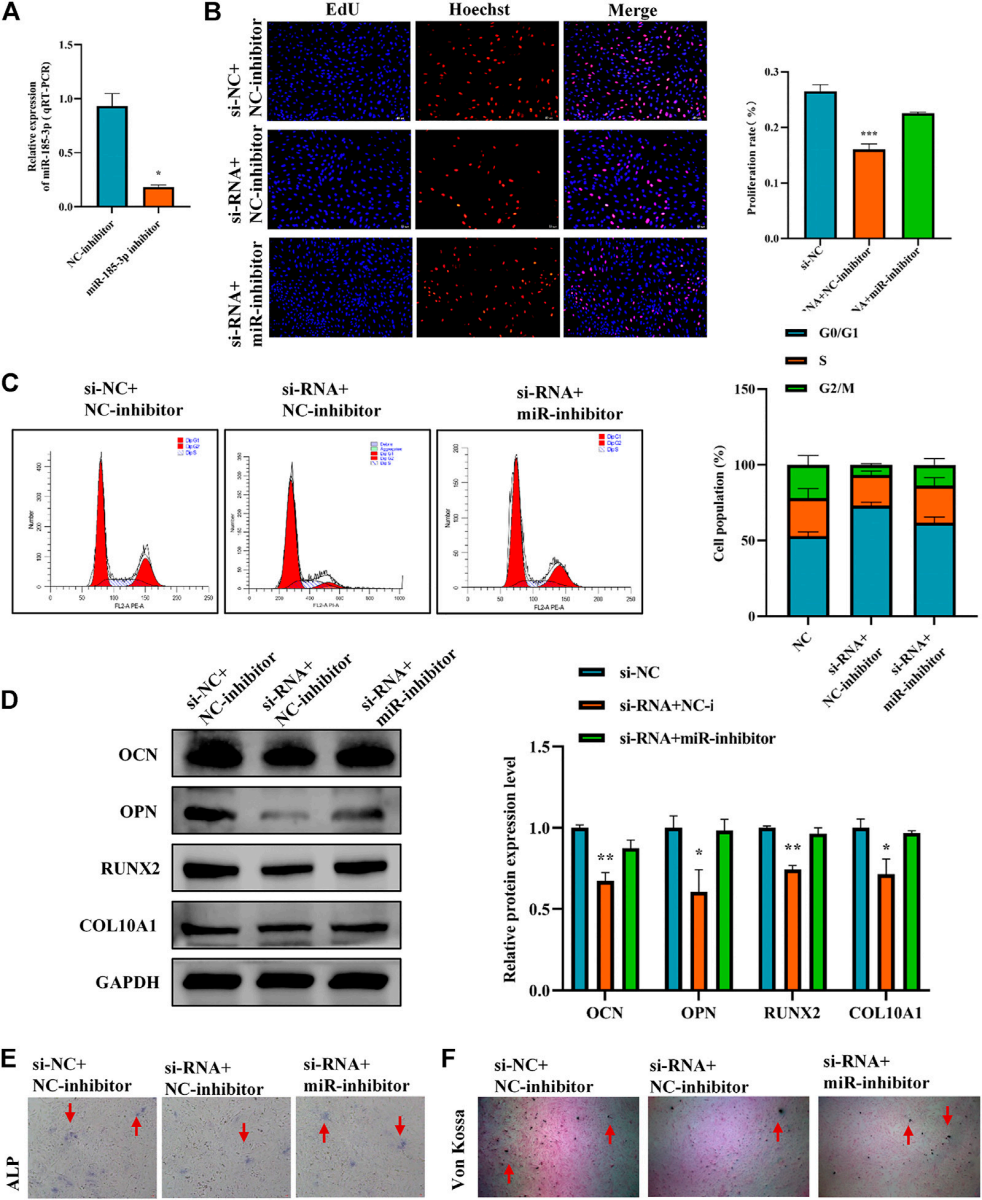
Hsa_circ_0008870 suppresses chondrocytes growth and proliferation and cartilage osteogenesis by targeted upregulation of miR-185-3p. **(A)** The RNA levels of miR-185-3p in human chondrocytes transfected with miR-185-3p inhibitor were measured by RT-qPCR. **(B)** Proliferation of chondrocytes downregulated by hsa_circ_0008870 was detected by Edu assays, with or without miR-185-3p inhibitor. **(C)** The effect of hsa_circ_0008870 downregulation on chondrocyte cell cycle was detected by flow cytometry, with or without miR-185-3p inhibitor. **(D)** The protein expressions of OCN, OPN, RUNX2 and collagen type X were measured in hsa_circ_0008870 knockdown chondrocytes, with or without miR-185-3p inhibitor. **(E)** ALP activity was measured in hsa_circ_0008870 knockdown chondrocytes, with or without miR-185-3p inhibitor. **(F)** Von Kossa staining demonstrated changes in mineralization in hsa_circ_0008870 knockdown chondrocytes, with or without miR-185-3p inhibitor. P > 0.05; *, *p* < 0.05; **, *p* < 0.01; ***, *p* < 0.001; ****, *p* < 0.0001.3.1.26.

To clarify the interaction between hsa_circ_0008870 and miR-185-3p, we transfected miR-185-3p inhibitor in chondrocytes and assessed the transfection efficiency of inhibitor by RT-qPCR (Figure 5A). The results of rescue experiments indicated that silencing miR-185-3p reversed the inhibition of knockout hsa_circ_0008870 on chondrocyte proliferation compared with the negative control group (Figures 5B,C). Western blot results revealed that although knockdown of hsa_circ_0008870 downregulated the expression levels of OCN, OPN, RUNX2 and collagen type X, silencing miR-185-3p restored their expression levels (Figure 5D). Furthermore, ALP activity and mineralized nodules were restored in si-circ-0008870+ miR-185-3p inhibitor group (Figures 5E,F). To summarize, hsa_circ_0008870 suppresses chondrocytes proliferation and cartilage osteogenesis by targeted upregulation of miR-185-3p.

### 3.4 The silencing of hsa_circ_0008870 suppresses chondrocytes proliferation, hypertrophy, as well as endochondral osteogenesis *via* miR-185-3p/MAPK1 axis

**FIGURE 6:**
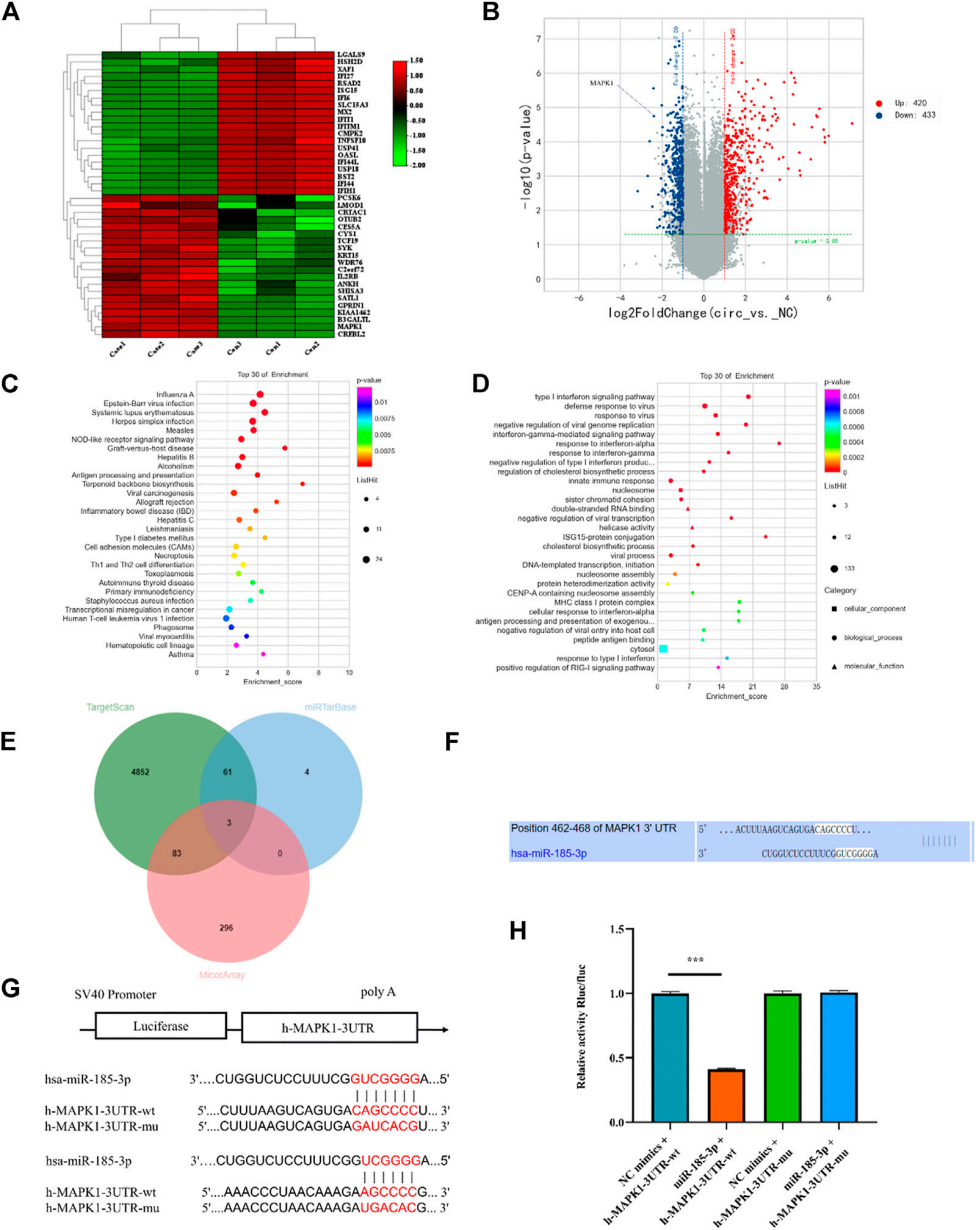
mRNA high-throughput sequencing and bioinformatics analyses following silencing hsa_circ_0008870, and luciferase activity between miR-185-3p and MAPK1. **(A)** The heatmap of differently expressed mRNAs in hsa_circ_0008870 knockdown chondrocytes (n = 3) and normal control chondrocytes (n = 3). **(B)** Volcano plot of the significantly upregulated and downregulated mRNAs. **(C,D)** The top 30 KEGG pathway and GO enrichment are exhibited. **(E)** Venn diagram demonstrating the intersection of downregulated mRNAs and predicted target mRNAs. **(F)** The potential target binding sites between miR-185-3p and MAPK1. **(G,H)** Luciferase activity was repressed in human chondrocyte cotransfected with WT MAPK1 and miR-185-3p mimics, while it was restored in cells cotransfected with Mut MAPK1 and miR-185-3p mimics. ***, *p* < 0.001.3.1.27.

**FIGURE 7:**
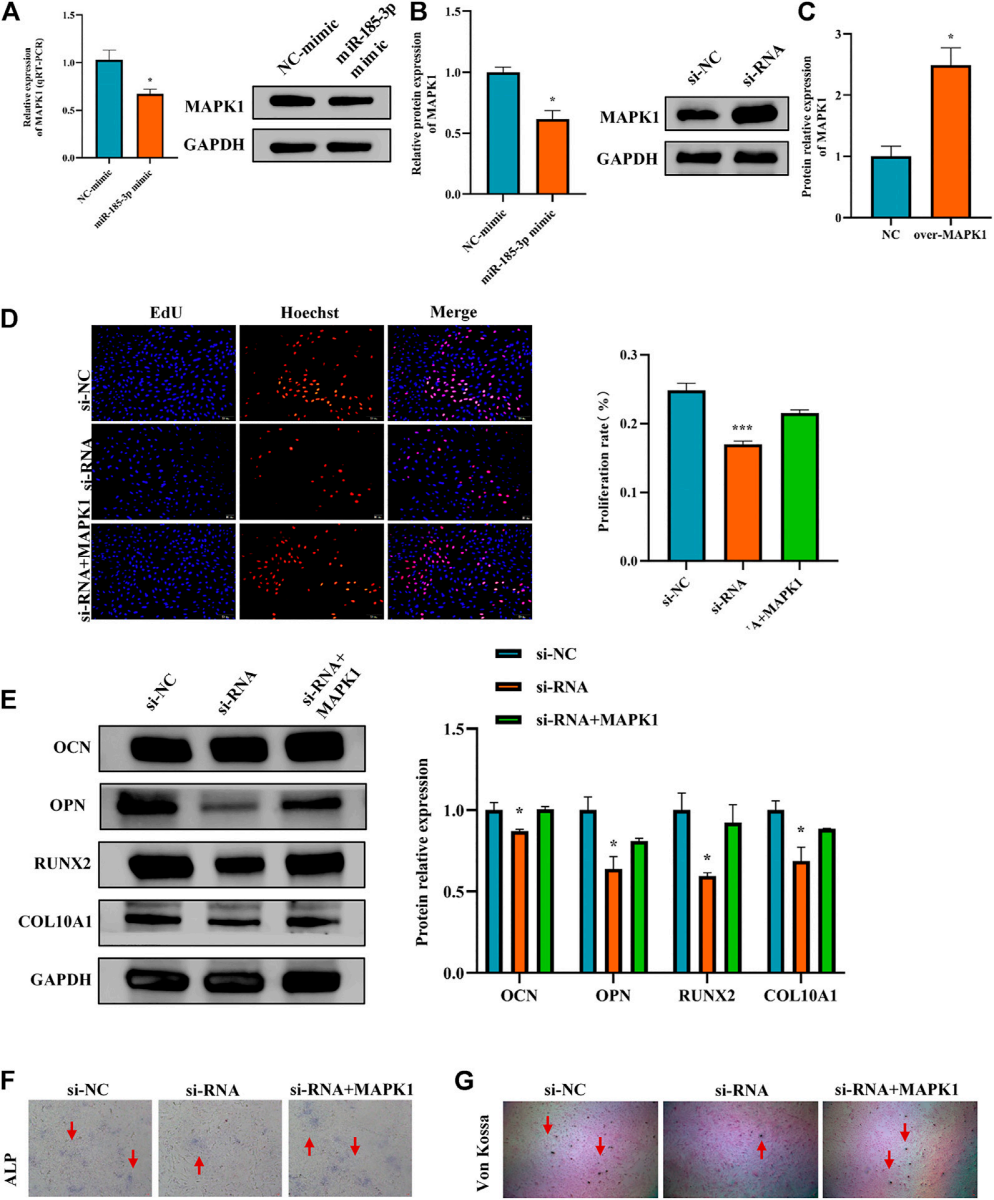
Hsa_circ_0008870 suppressed chondrocyte proliferation, hypertrophy and endochondral ossification by regulating miR-185-3p/MAPK1. **(A,B)** The mRNA and protein expression of MAPK1 was assessed with miR-185-3p mimics. **(C)** The protein expression of MAPK1 expression levels was assessed following overexpressing MAPK1. **(D)** The effect of MAPK1 overexpression on hsa_circ_0008870 knockdown-mediated cell proliferation was measured by Edu assays. **(E)** The protein expressions of OCN, OPN, RUNX2 and collagen type X were analyzed by Western blot. **(F,G)** The effect of MAPK1 overexpression on hsa_circ_0008870 knockdown-mediated ALP activity and Von Kossa staining. *, *p* < 0.05 and ***, *p* < 0.001.3.1.28.

To determine the downstream gene of miR-185-3p, the differential expression of mRNAs was screened by high-throughput sequencing analysis after silencing of the hsa_circ_0008870 in human chondrocytes (Figures 6A,B). The result show that 853 differentially expressed mRNAs were recognized, 433 of them were downregulated whereas the rest 420 were upregulated (*p* < 0.05, |logFC|-value > 2.0). The top 30 KEGG pathways and GO enrichment analyses were summarized in Figures 6C,D. In addition, we predicted potential downstream target genes of miR-185-3p using two online databases (TargetScan and miRTarBase). According to the obtained Venn diagram (Figure 6E), there are three genes in common. According to the high-throughput sequencing analysis results, the differential expression fold of MAPK1 ranks among the top ten, and studies have confirmed that MAPK1 can maintain the metabolism and homeostasis of chondrocytes, thus playing a protective role (Feng et al., 2010). Therefore, MAPK1 was selected as the target gene of miR-185-3p. The binding sites of miR-185-3p and MAPK1 are shown in Figures 6F,G. The expression levels of MAPK1 was significantly downregulated by upregulating the expression of miR-185-3p (Figures 7A,B.

The Luciferase reporter assay indicated that the relative luciferase activity of miR-185-3p + MAPK1 3′UTR-wt was significantly inhibited, while the relative luciferase activity of miR-185-3p + MAPK1 3′UTR-mut group had no significant change (Figures 6G,H). Previous studies had shown that MAPK1 not only promoted the proliferation, growth and differentiation of chondrocytes but also stimulated the formation of osteoblasts in the process of endochondral osteogenesis. To further evaluate whether hsa_circ_0008870 regulated the progress of ISS by regulating the expression levels of MAPK1, we carried rescue experiments. The transfection of MAPK1 protein in chondrocytes significantly promoted the expression levels of MAPK1, confirming the successful transfection of MAPK1 protein (Figure 7C). Transfected human chondrocytes with both si-circ-0008870 and MAPK1 protein showed that the si-circ-0008870 + MAPK1 protein group reversed the inhibition of hsa_circ_0008870 knockdown on chondrocyte proliferation (Figure 7D). Function experiments manifested that the inhibitory effect of hsa_circ_0008870 knockdown on the proliferation and hypertrophy of chondrocytes was reversed by adding MAPK1 protein. Consistently, the inhibition of hsa_circ_0008870 knockdown on the expression levels of OCN, OPN, RUNX2, and collagen type X proteins was suppressed by the overexpression of MAPK1 (Figure 7E). This was indicated by the increase of the number of mineralized nodules and ALP activity in the silenced hsa_circ_0008870 after the overexpression of MAPK1 (Figures 7F,G). The *in vitro* experimental results showed that hsa_circ_0008870 knockdown inhibited chondrocytes proliferation, hypertrophy, as well as endochondral osteogenesis *via* miR-185-3p/MAPK1 axis.

### 3.5 Upregulation of hsa_circ_0008870 enhanced chondrocytes hypertrophy and endochondral osteogenesis by regulating miR-185-3p/MAPK1

**FIGURE 8:**
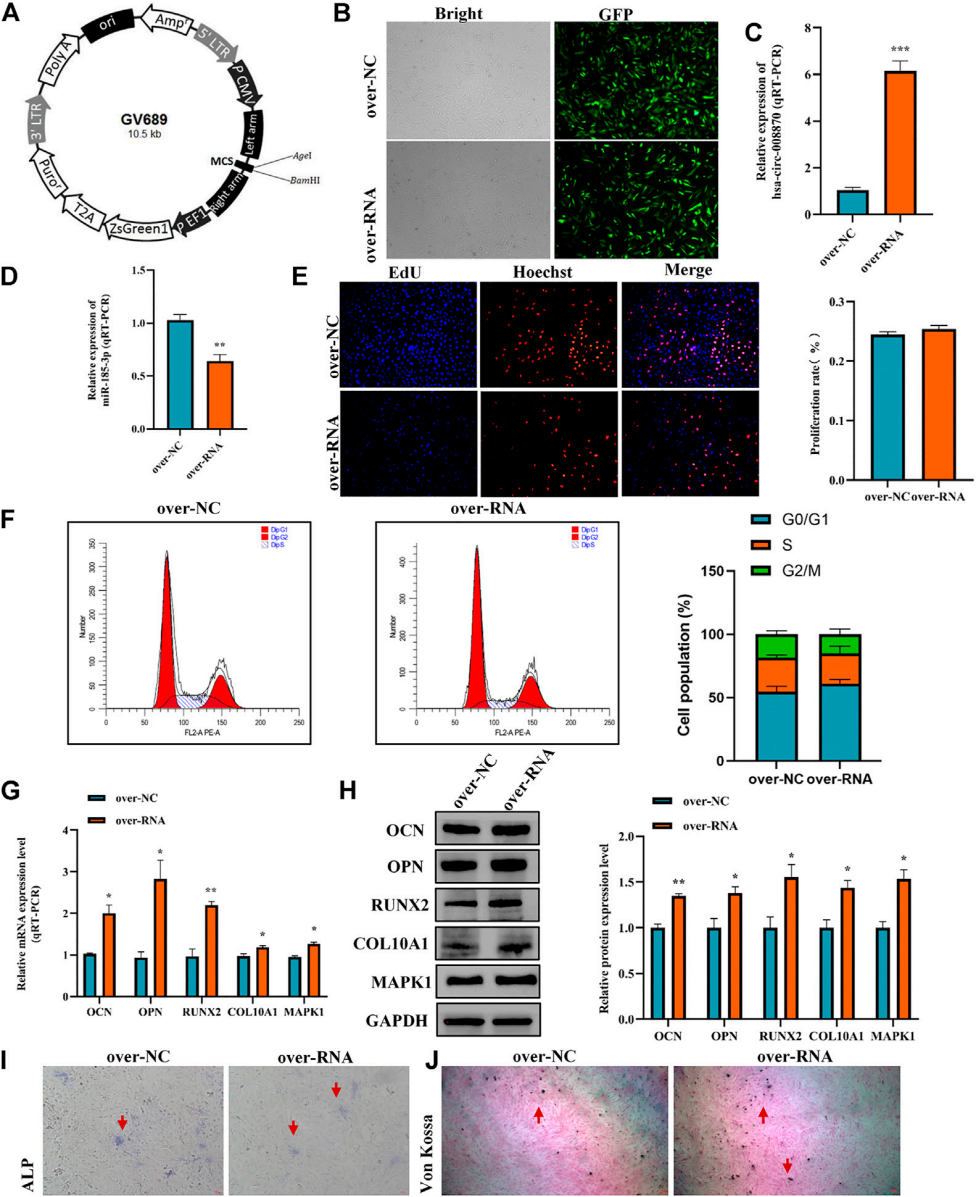
Overexpression of hsa_circ_0008870 promoted endochondral osteogenesis, hypertrophy and the growth and proliferation of chondrocytes by regulating miR-185-3p/MAPK1. **(A)** Hsa_circ_0008870 overexpressing lentivirus. **(B)** Efficiency of lentiviral transduction were detected by fluorescence of green fluorescent protein. **(C,D)** The RNA levels of hsa_circ_0008870 and miR-185-3p in human chondrocytes transfected with over_circ_0008870 lentivirus were measured by RT-qPCR. **(E)** The effect of overexpression of hsa_circ_0008870 on cell proliferation was measured by Edu assays. **(F)** The effect of overexpression of hsa_circ_0008870 on cell cycle was detected by flow cytometry assays. **(G,H)** The expressions of OCN, OPN, RUNX2, collagen type X and MAPK1 were analyzed by RT-qPCR and Western blot. **(I,J)** The effect of hsa_circ_0008870 overexpression on ALP activity and Von Kossa staining. *p < 0.05, **p < 0.01 and ***p < 0.001.3.1.29.

To further reveal the effect of hsa_circ_0008870 in chondrocytes proliferation, hypertrophy, as well as endochondral osteogenesis, we first constructed hsa_circ_0008870 overexpressing lentivirus (Figure 8A) and the hsa_circ_0008870 was successfully transfected and overexpressed in human chondrocytes (Figures 8B,C). Subsequent results revealed that miR-185-3p was downregulated (Figure 8D). Although there was no significant difference in the proliferation of chondrocyte between the hsa_circ_0008870 over-expression and the control groups (Figures 8E,F) *via* Edu assays and flow cytometry analysis, the expression of OCN, OPN, RUNX2, collagen type X and MAPK1 were significantly upregulated in hsa_circ_0008870 over-expression group (Figures 8G,H). In addition, the ALP active nodules and mineralized nodules were promoted (Figures 8I,J). These results indicated that the upregulated hsa_circ_0008870 promoted chondrocytes hypertrophy and endochondral osteogenesis.

### 3.6 The miR-185-3p upregulation can inhibit the expression of hsa_circ_0008870 in chondrocytes *via* a positive feedback loop

**FIGURE 9:**
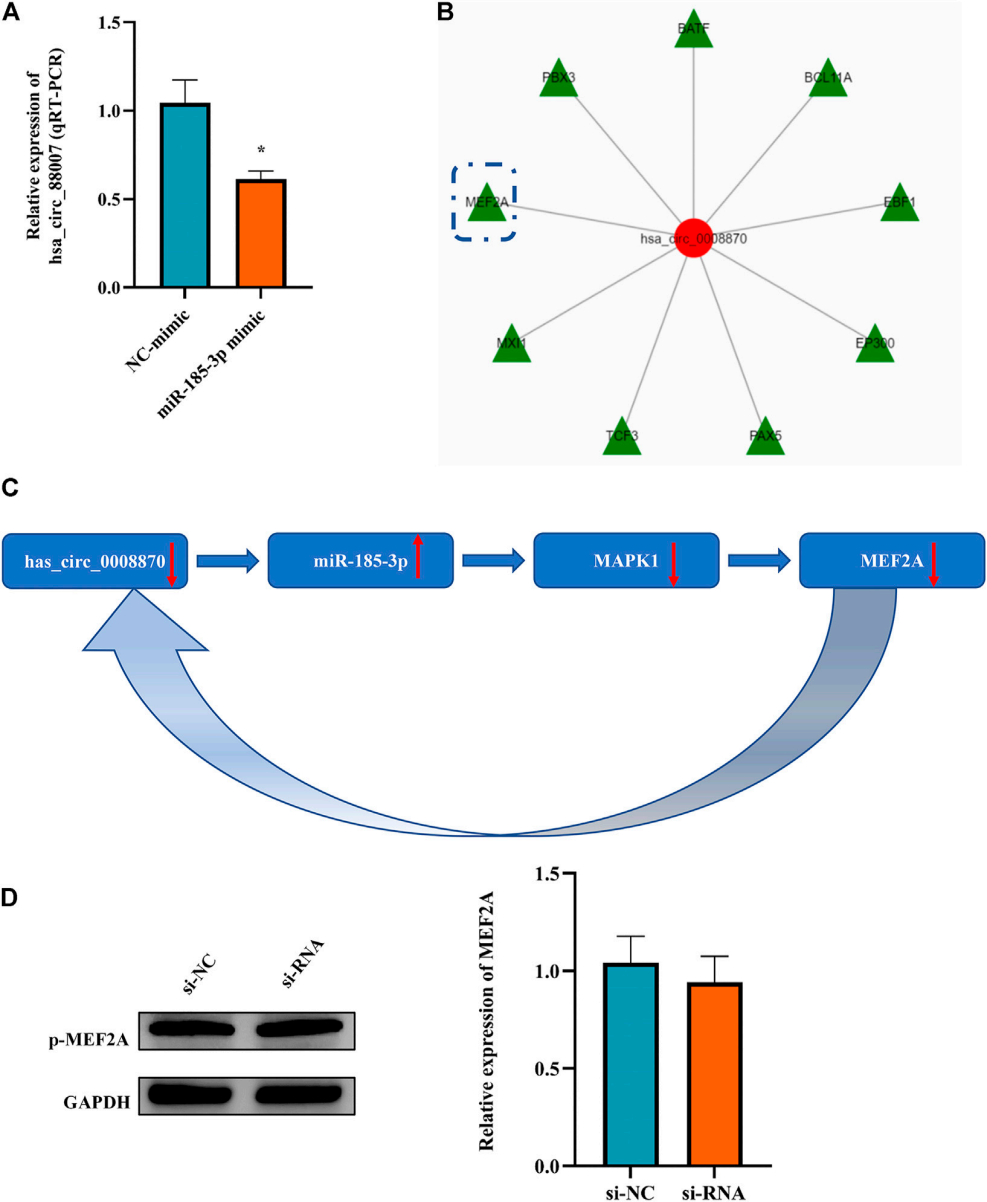
The miR-185-3p upregulation can inhibit the expression of hsa_circ_0008870 in chondrocytes via a positive feedback loop. **(A)** The expression of hsa_circ_0008870 was assessed with miR-185-3p mimics. **(B)** Transcription factors of hsa_circ_0008870 were analyzed by bioinformatics. **(C)** The summary of the positive feedback regulation of hsa_circ_0008870/miR-185-3p/MAPK1/MEF2A loop. **(D)** The protein expressions of phosphorylated MEF2A after knockdown of hsa_circ_0008870 were detected by Western blot. *p < 0.05.3.1.30.

It is an interesting phenomenon that we observed that not only hsa_circ_0008870 can regulate the expression of miR-185-3p *via* the sponge mechanism, but also the upregulated miR-185-3p could inhibit the expression of hsa_circ_0008870 (Figure 9A). For example, as shown Figure 5, the silencing of hsa_circ_0008870 inhibited chondrocytes proliferation and endochondral osteogenesis *via* upregulating miR-185-3p. Subsequently, it was observed that the expression of MAPK1 and hsa_circ_0008870 were downregulated when miR-185-3p was upregulated in human chondrocytes. Hence, we hypothesized that there might be a positive feedback loop underlying the co-regulation between hsa_circ_0008870 and miR-185-3p in ISS. Bioinformatics analysis demonstrated that the MEF2A is the transcription factor of hsa_circ_0008870 (Figure 9B). Meanwhile, several studies have suggested that MAPK1 can downregulate the expression of MEF2. And there are four mammalian MEF2 genes, MEF2A, -B, -C, and -D (McKinsey et al., 2002). Therefore, we speculate that upregulated miR-185-3p suppresses the expression of MAPK1, which induces the downregulation of MEF2A expression. As a result, hsa_circ_0008870 expression was inhibited (Figure 9C). Though our results observed that phosphorylated active of MEF2A was inhibited after upregulation of miR-185-3p, no statistical significance was shown (Figure 9D). Unfortunately, our experiments do not reveal the regulation mechanism of positive feedback loop between miR-185-3p and hsa_circ_0008870.

### 3.7 *In vivo* experiment further verified that hsa_circ_0008870 inhibited proliferation, hypertrophy and bone formation of growth plate *via* miR-185-3p/MAPK1 axis

**FIGURE 10:**
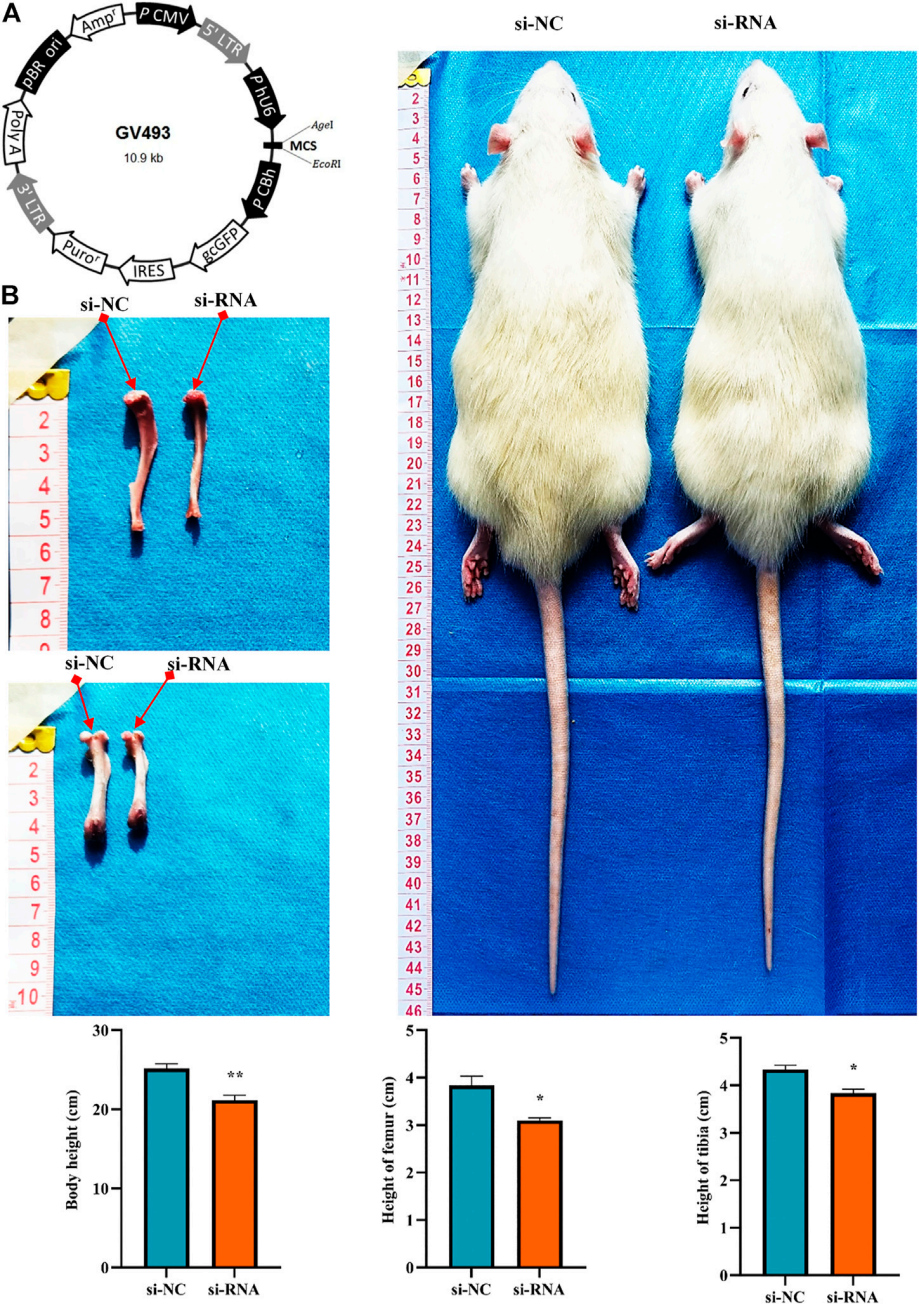
Knockdown of hsa_circ_0008870 resulted in smaller stature in rats. **(A)** Hsa_circ_0008870 knockdown lentivirus. **(B)** Compared with the rats in the negative control group, changes in body size, tibia and femur length of hsa_circ_0008870 knockdown rats. *p < 0.05 and **p < 0.01.3.1.31.

**FIGURE 11:**
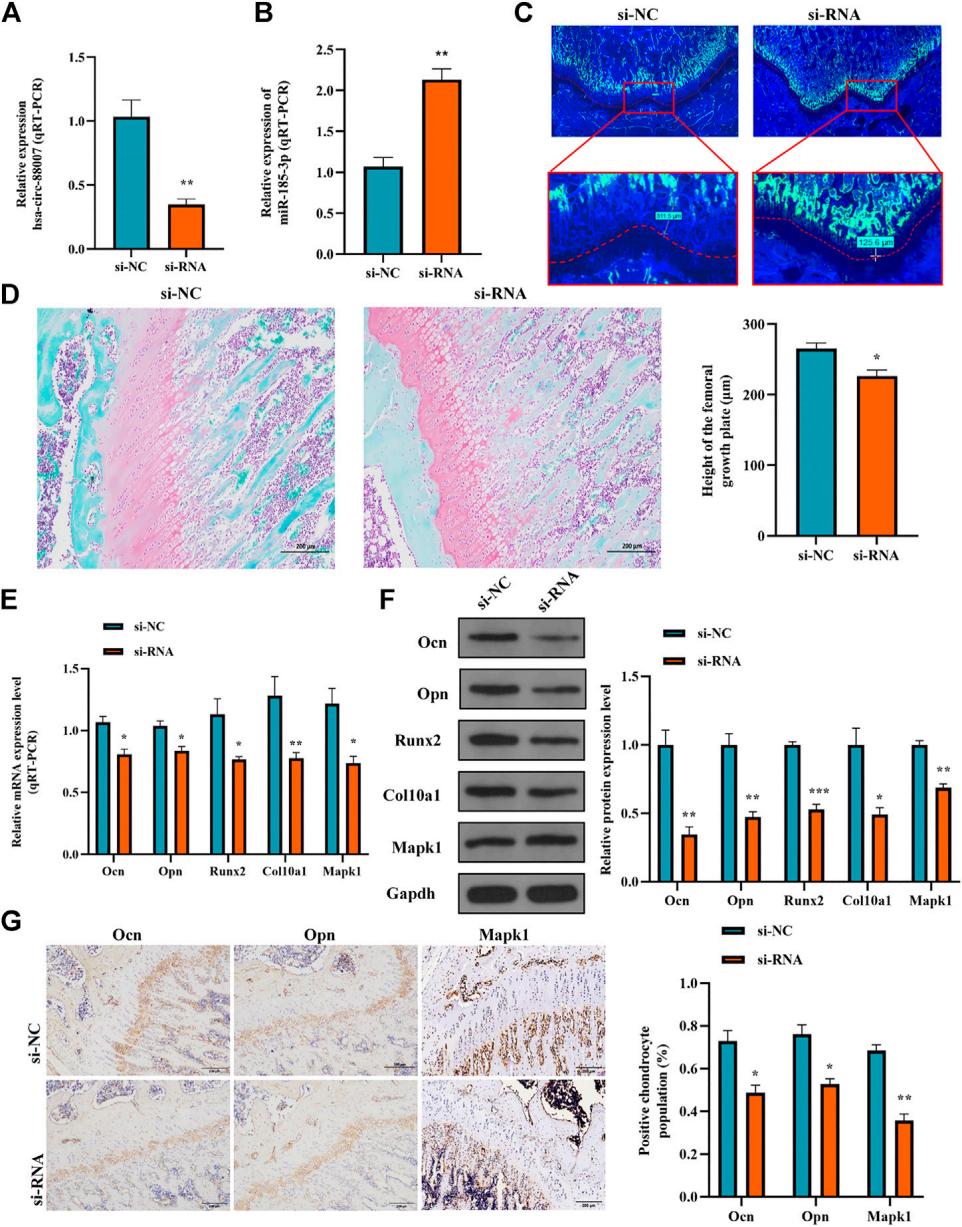
Hsa_circ_0008870/miR-185-3p/MAPK1 axis inhibits endochondral osteogenesis as well as the hypertrophy, proliferation and growth of chondrocytes *in vivo*. **(A,B)** The RNA levels of hsa_circ_0008870 and miR-185-3p in rats injected with si-circ-0008870 or normal control lentivirus were measured by RT-qPCR. **(C)** Analysis of the new bone formation rate in the rats by calcein labeling. **(D)** Assessment of the height of the femoral Growth plate in rats by Safranin O staining. **(E,F)** The mRNA and protein expressions of Ocn, Opn, Runx2, collagen type X and Mapk1 were detected by RT-qPCR. **(G)** Expression levels of Ocn, Opn and Mapk1 in rat growth plates were analyzed by immunohistochemical staining. *p < 0.05, **p < 0.01 and ***p < 0.001.

To clarify the effect of the hsa_circ_0008870 knockdown on the rat’s stature, a lentivirus carrying si-circ-0008870 was constructed and used to downregulate the expression of hsa_circ_0008870 in wild-type rats *via* tail vein (Figure 10A). Compared with the rats in the negative control group, the body size, tibia and femur length of the hsa_circ_0008870 knockdown rats were reduced (Figure 10B). RT-qPCR results manifested that hsa_circ_0008870 was upregulated in rat growth plate cartilage, and miR-185-3p was downregulated after downregulation of hsa_circ_0008870 expression (Figures 11A, 1B). Calcein staining revealed that the rate of new bone formation was lower in the rats of hsa_circ_0008870 downregulation than that in the control rats (Figure 11C). Safranin O staining demonstrated that the height of the femoral growth plate in the rats of hsa_circ_0008870 downregulation was significantly shorter compared to control rats (Figure 11D).

Afterwards, the mRNA and protein expression levels of Ocn, Opn, Runx2, the collagen type-X and Mapk1 in rat growth plates were determined by RT-qPCR and Western blot (Figures 11E, 1F). Compared to control rats, the expression of Ocn, Opn, Runx2 collagen type X and Mapk1 genes were significantly downregulated in the rats of hsa_circ_0008870 downregulation. The immunohistochemical analysis indicated that the expression of Ocn, Opn and Mapk1 were also markedly downregulated in the rats of hsa_circ_0008870 downregulation (Figure 11G). To sum up the above, these results demonstrate that knock down the expression of hsa_circ_0008870 damages the proliferation and bone formation of growth plate in rats. As a result, the rats showed a short stature phenotype.

## 4 Discussion

Circular RNAs are found in stable forms in human peripheral blood, according to recent studies, and the expression of circRNAs in blood may contain disease-related information (Memczak et al., 2015; Zhang et al., 2020; Yang et al., 2022). For example, Yang et al. discovered that the patients with ischemic stroke had significantly reduced expression of circUSP36 in their peripheral blood, and it alleviated the injury of ischemic stroke injury *via* miR-139-3p/SMAD3 axis (Yang et al., 2022). circPPM1F is evidently upregulated in peripheral blood of children with type 1 diabetes mellitus, and overexpression of circPPM1F can promote islet injury by enhancing the activation of M1 macrophages (Zhang et al., 2020). In our study, authors first confirmed that hsa_circ_0008870 was downregulated in ISS patients. However, to date, the biological function of hsa_circ_0008870 has not been addressed in the literature published.

In order to determine whether hsa_circ_0008870 is the cause or result of ISS, the authors down regulated the expression of hsa_circ_0008870 in human chondrocytes. These experiments outcomes demonstrated that hsa_circ_0008870 knockdown inhibited chondrocytes proliferation, hypertrophy, and endochondral ossification, whereas overexpression of hsa_circ_0008870 had the opposite results. This suggests that hsa_circ_0008870 may play a key role on ISS pathogenesis. Subsequent *in situ* hybridization experiment showed hsa_circ_0008870 was localized in the cytoplasm, which indicates that hsa_circ_0008870 could function as miRNA sponges. The targeted miRNAs of hsa_circ_0008870 were predicted by bioinformatics analysis and validity was further assessed by RT-qPCR after hsa_circ _0008870 silencing. MiR-185-3p presented the greatest differential expression in chondrocytes after hsa_circ_0008870 silencing. Thus, it was selected as a candidate target microRNA for further study in the present study. Previous studies observed that overexpression of miR-185-3p improved the renal function of diabetic nephropathy mice by inhibiting and reducing the expression of AGER (Xue et al., 2020). Researchers found that miR-185-3p overexpression enhanced the chemical sensitivity of drug-resistant colorectal cancer (CRC) cells through inhibiting AQP5, offering potential therapeutic targets for CRC cells that are resistant to 5-FU (Zhou et al., 2020). Li et al. demonstrated that downregulated miR-185-3p regulated WNT2B expression *in vitro* inhibiting the proliferation and transfer of nasopharyngeal carcinoma cells (Li C et al., 2021). However, few study explained the role of miR-185-3p on chondrocytes and growth plates.

To further identify the biological function of miR-185-3p on chondrocytes, the authors have applied fluorescence *in situ* hybridization. These outcomes showed that hsa_circ_0008870 and miR-185-3p expression co-localized in human chondrocytes and femur growth plates of SD rats. Upregulation of miR-185-3p suppressed the proliferation and hypertrophy, and endochondral osteogenesis in chondrocytes. Subsequent experiments observed that these phenotypes caused by has_circ_0008870 downregulated were rescued *via* silencing miR-185-3p in chondrocytes. The dual-luciferase reporter gene experiment further confirmed that hsa_circ_0008870 negatively regulated miR-185-3p expression. The results suggested that hsa_circ_0008870 mediated the progression of ISS *via* targeting miR-185-3p.

To verify the targeted genes of miR-185-3p, the differentially expressed mRNA in human chondrocytes was screened by high-throughput sequencing analysis after silencing of hsa_circ_0008870. According to these results, the top six mRNAs were screened. Moreover, the targeted mRNA of miR-185-3p was selected by bioinformatic analysis. Among all overlap mRNAs between the top six mRNAs and bioinformatic analysis, MAPK1 has attracted attention as it can promote chondrocyte proliferation and alleviate chondrocytes damage. For example, Li et al. reported that the inflammation damage of chondrocytes induced by IL-1 was improved by MAPK1 upregulation (Li Q et al., 2021). Zhao et al. indicated that overexpression of MAPK1 reduced the inhibitory effect of miR-320c on articular chondrocyte proliferation and attenuated the promoting effect of miR-320c on apoptosis (Zhao et al., 2021). Wang et al. observed that the positive feedback regulation between ERK2 (MAPK1) and USP15 played a critical role in the regulation of TGF-β/SMAD2 signaling and the maintaining of cartilage phenotype (Wang et al., 2021).

In the present study, the authors observed that miR-185-3p overexpression significantly suppressed the expression of MAPK1. Meanwhile, luciferase reporter assay showed that miR-185-3p can target MAPK1. To further confirm the biological role of MAPK1 in hsa_circ_0008870/miR-185-3p, the rescue experiments were carried out. As predicted, the over-regulation of MAPK1 reversed the inhibition of the proliferation and hypertrophy of chondrocytes and endochondral osteogenesis caused by the downregulation of hsa_circ_0008870. Meanwhile, we also found that hsa_circ_ 0008870 overexpression can enhance chondrocyte hypertrophy and endochondral ossification. These outcomes indicated that hsa_circ_0008870 can regulate the proliferation and hypertrophy of chondrocytes and endochondral osteogenesis *via* the miR-185-3p/MAPK1 axis. Subsequently, the effect of the hsa_circ_0008870/miR-185-3p/MAPK1 axis in the progress of ISS was further confirmed *in vivo*.

In the present study, the authors also observed an interesting phenomenon that upregulated of miR-185-3p can in turn inhibit hsa_circ_0008870 expression in chondrocytes. The upstream transcription factors of hsa_circ_0008870 was screened *via* UCSC analysis. The outcomes demonstrated that MEF2A might be a transcription factor binding to hsa_circ_0008870 promoter sequences. There are four mammalian MEF2 genes, MEF2A, -B, -C and -D (McKinsey et al., 2002). Rampalli et al. reported P38 MAPK kinase regulates transcription by phosphorylation of myocyte enhancer factor-2 (MEF2) protein to recruit methylase to MEF2 target gene for epigenetic modification of its target gene (Rampalli et al., 2007). Previous studies reported that one of the most typical targets of MAPK1 was the transcription factor of MEF2 family (Yang et al., 1998; Fukuhara et al., 2000; Kato et al., 2000). MAPK1 increased the phosphorylation of MEF2 promoting the activation of MEF2 (Olson, 2004). To further analyze whether MAPK1 influences MEF2A gene expression by its phosphorylation in our study, the expression of phosphorylated MEF2A was detected after silencing hsa_circ_0008870. These results revealed that phosphorylated active of MEF2A was inhibited, while there was no statistical significance. The current study still has several limitations. To date, the underlying mechanism of positive feedback loop between hsa_circ_0008870 and miR-185-3p is still uncertain and requires further attention in future study. Additionally, though no significant difference in sex distribution was observed between the ISS and normal children using Chi square test (*p* = 0.175), our samples present a discordant sex distribution. Multi center research may be beneficial to further confirm and refute our findings.

In conclusions, our study revealed a novel role of hsa_circ_0008870 in the progression of ISS through miR-185-3p/MAPK1 axis. This suggests the hsa_circ_0008870 might be a potential biomarker and new therapeutic target for ISS.

## Data Availability

The original contributions presented in the study are included in the article/Supplementary Material, further inquiries can be directed to the corresponding authors.
