# Supplementary material for: Hsa_circ_0008870 suppresses bone formation of growth plate through inhibition of miR-185-3p/ MAPK1 axis in idiopathic short stature
Source: Front Bioeng Biotechnol. 2022 Oct 11;10:1022830. doi: 10.3389/fbioe.2022.1022830 (PMC9592914; doi:10.3389/fbioe.2022.1022830)
Supplement: Supplementary file 1 [file Table1.docx]

Supplementary Table 1 Primers used for qRT- PCR analysis of circRNA, miRNA and mRNA levels

| Target ID | Forward (5'-3') | Reverse (5'-3') |
| --- | --- | --- |
| has_circ_0008870 | GACCTACTGCCAGAGAAC | GACCTACTGCCAGAGAAC |
| miR-185-3p | GGGGCTGGCTTTCCTCTG | GTGGAGTCGGCAATTGCAC |
| miR-616-3p | ACACTCCAGCTGGGAGTCATTGGAGGGTTT | TGGTGTCGTGGAGTCG |
| miR-597-3p | ACACTCCAGGTCGGGTGGT | ATTGGAACGATACAGAGAAGATT |
| miR-29a-3p | CTGCCGTAGCACCATCTGA | TATCCTTGTTCACGAACTCCTTCAC |
| miR-335-3p | TTTTTCATTATTGCTC | GTGCAGGGTCCGAGGT |
| miR-326 | GCCGAGCCTCTGGGCCCTTC | CAGTGCGTGTGTGTGGAGT |
| miR-330-5p | TCTGGGCCTGTGTCTTAGGC | GCTATCTCAGGGTGTGTGGTGGTGGG |
| miR-487a | GCGGCGGAATCATACAGGGACATC | ATCCAGTGCAGGGTCCGAGG |
| miR-513a-5p | TAAATTTCACCTTTCTGAGAAGG | GCGAGCACAGAATTAATACGAC |
| miR-526b | GCGACTCTTGAGGGAAGCACT | AGTGCAGGGTCCGAGGTATT |
| miR-578 | GTGCAGGGTGTTAGGA | GAAGAACGTCTGGT |
| miR-579 | GTGCAGGGTCCGAGGT | TTAACAAAGTG CTCATAGTGC |
| miR-587 | CCAGGCAAGAGAGTTGCTG | AGTCACAGGTGCAGACACATT |
| miR−599 | GTTGTGTCAGTTTA | CAGTGCGTGTGTGTGGAGT |
| miR-653 | GTGTTGAAACAATCT | GTGCAGGGTCCGAGGT |
| miR-654-3p | GGGATGTCTGCTGACCA | CAGTGCGTGTCGTGGA |
| U6 | CTCGCTTCGGCAGCACA | AACGCTTCACGAATTTGCGT |
| MAPK1 | GCACCAACCATCGAGCAAAT | CTTGAGGTCACGGTGCAGAA |
| OCN | GCACCACCGTTTAGGGCAT | CGTTCCTCATCTGGACTTTATTTTG |
| OPN | CCAGCCAAGGACCAACTACA | AGTGTTTGCTGTAATGCGCC |
| RUNX2 | ACTTCCTGTGCTCCGTGCTG | TCGTTGAACCTGGCTACTTGG |
| COL10A1 | GCAGCATTACGACCCAAGAT | CATGATTGAACTCCCTGAAG |
| GAPDH | GGAGCGAGATCCCTCCAAAAT | GGCTGTTGTCATACTTCTCATGG |
| Ocn | GCAGACCTAGCAGACACCAT | GCAGACCTAGCAGACACCAT |
| Opn | CAGCAGCAGGACTGAAGGA | CAGCAGCAGGACTGAAGGA |
| Runx2 | AACCAAGTGGCCAGGTTCAA | GGACCGTCCACTGTCACTTT |
| Col10a1 | CCAGCCAAGCAGTCATACCT | CCAGCCAAGCAGTCATACCT |
| Gapdh | GCTGAGTATGTCGTGGAGTCT | GCTGAGTATGTCGTGGAGTCT |
